# Chemically Modified DNA Aptamers Bind Interleukin-6 with High Affinity and Inhibit Signaling by Blocking Its Interaction with Interleukin-6 Receptor

**DOI:** 10.1074/jbc.M113.532580

**Published:** 2014-01-12

**Authors:** Shashi Gupta, Masao Hirota, Sheela M. Waugh, Ikuo Murakami, Tomoki Suzuki, Masahiro Muraguchi, Masafumi Shibamori, Yuichi Ishikawa, Thale C. Jarvis, Jeffrey D. Carter, Chi Zhang, Bharat Gawande, Michael Vrkljan, Nebojsa Janjic, Daniel J. Schneider

**Affiliations:** From ‡ SomaLogic, Inc., Boulder, Colorado 80301 and; the § Otsuka Pharmaceutical Co., Ltd., 463-10 Kagasuno, Kawauchi-cho, Tokushima 771-0192, Japan

**Keywords:** Aptamers, Cell Signaling, Drug Discovery, Interleukin, Molecular Evolution, Nucleic Acid Chemistry, Protein-DNA Interaction, SELEX, SOMAmer, Nuclease Stability

## Abstract

Interleukin-6 (IL-6) is a pleiotropic cytokine that regulates immune and inflammatory responses, and its overproduction is a hallmark of inflammatory diseases. Inhibition of IL-6 signaling with the anti-IL-6 receptor antibody tocilizumab has provided some clinical benefit to patients; however, direct cytokine inhibition may be a more effective option. We used the systematic evolution of ligands by exponential enrichment (SELEX) process to discover slow off-rate modified aptamers (SOMAmers) with hydrophobic base modifications that inhibit IL-6 signaling *in vitro*. Two classes of IL-6 SOMAmers were isolated from modified DNA libraries containing 40 random positions and either 5-(*N*-benzylcarboxamide)-2′-deoxyuridine (Bn-dU) or 5-[*N*-(1-naphthylmethyl)carboxamide]-2′-deoxyuridine (Nap-dU) replacing dT. These modifications facilitate the high affinity binding interaction with IL-6 and provide resistance against degradation by serum endonucleases. Post-SELEX optimization of one Bn-dU and one Nap-dU SOMAmer led to improvements in IL-6 binding (10-fold) and inhibition activity (greater than 20-fold), resulting in lead SOMAmers with sub-nanomolar affinity (*K_d_* = 0.2 nm) and potency (IC_50_ = 0.2 nm). Although similar in inhibition properties, the two SOMAmers have unique sequences and different ortholog specificities. Furthermore, these SOMAmers were stable in human serum *in vitro* for more than 48 h. Both SOMAmers prevented IL-6 signaling by blocking the interaction of IL-6 with its receptor and inhibited the proliferation of tumor cells *in vitro* as effectively as tocilizumab. This new class of IL-6 inhibitor may be an effective therapeutic alternative for patients suffering from inflammatory diseases.

## Teaser

**Background:** IL-6 signaling is a key component of inflammatory diseases.

**Results:** Modified DNA aptamers that inhibit IL-6 signaling were discovered and optimized.

**Conclusion:** Modified aptamers are stable in serum and block the interaction of IL-6 with its receptor IL-6Rα.

**Significance:** Modified aptamers are a new class of antagonist with properties potentially suitable for clinical treatment of inflammation.

## Introduction

IL-6 is a member of the cytokine family of immunomodulating proteins, characterized by a long chain four-helix bundle (1–3). IL-6 is produced by B cells, T cells, monocytes, fibroblasts, and other cell types and exhibits both pro- and anti-inflammatory properties (4, 5). IL-6 activates cells by binding to its specific nonsignaling IL-6 receptor (IL-6Rα, gp80, and CD126) present on the cell membrane. This ligand-receptor complex then binds to the signal-transducing protein gp130 (CD130) and activates the JAK-STAT3-signaling pathway (1). IL-6Rα is expressed as a membrane-bound protein in only a few cell types, whereas gp130 is expressed ubiquitously in all cell types and acts as a signaling protein for other members of the IL-6 cytokine family. IL-6 signaling through membrane-bound IL-6Rα is known as the classical signaling pathway or cis-signaling. In addition to the membrane-bound IL-6Rα, a soluble form of IL-6Rα (sIL-6Rα) is present in high concentration in blood and other body fluids (6, 7) and has an affinity for IL-6 that is similar to the membrane-bound receptor. Upon interaction with IL-6, sIL-6Rα does not act as an antagonist; instead it increases the circulating half-life of IL-6 and activates the signaling pathway in cells where the membrane-bound form of IL-6Rα is not expressed. This is also known as the trans-signaling pathway (8, 9). The ubiquitous expression of gp130 suggests that the IL-6 trans-signaling pathway can activate all or most of the cell types in the body. A soluble form of gp130 is also expressed in cells and acts as an antagonist for the IL-6-signaling pathway. Thus, the different forms of IL-6Rα and gp130 play a role in regulating IL-6-mediated pathways in different cell types.

IL-6 is a pleiotropic regulator of a wide range of biological activities, including host immune defense mechanisms and hematopoiesis. It is also involved in the proliferation and differentiation of various tumor cells (10). Under some acute inflammatory conditions, IL-6 concentrations in plasma can dramatically increase from picograms/ml to micrograms/ml (11). The role of cytokines and their receptors in various inflammatory diseases has been elucidated in preclinical studies, and some have become major therapeutic targets (12, 13). There are now several available anti-TNF-α agents (such as infliximab, adalimumab, etanercept, golimumab, and centolizumab pegol) that are broadly used to reduce inflammation. Because these drugs are not effective in all patients, there is a need to explore other cytokines as targets for therapeutic intervention in inflammation, such as IL-6. Anti-IL-6Rα antibody tocilizumab was the first antagonist of the IL-6-signaling pathway to receive regulatory approval and is currently used for treating rheumatoid arthritis (14–16). Tocilizumab has also been tested in clinical trials for various other diseases (17), and several other antagonists of the IL-6 pathway are in development, including direct inhibitors of IL-6 (12). Although treatment options for inflammatory diseases have improved over the last several decades, there is still a need for alternative interventions for patients that do not respond to current therapies.

We report the discovery of novel aptamer-based antagonists of IL-6. Aptamers are oligonucleotides that bind their targets with high affinity and specificity and are selected by the process of systematic evolution of ligands by exponential enrichment (SELEX)^2^ (18, 19). Aptamers have been used for a wide range of both *in vitro* and *in vivo* applications, including affinity chromatography, image microscopy, and biomarker identification (20–22). With one approved drug, pegaptanib (Macugen) (23, 24), and several in clinical development (such as REG1 (25), E10030 (Fovista) (26), and ARC1905 (27)), aptamers are of increasing interest as therapeutic agents. Aptamers have a relatively small size (6–12 kDa) and therefore good diffusibility, low immunogenicity, and tunable binding and pharmacokinetic properties (28, 29), and they may represent a superior treatment option for certain indications.

We recently described a new class of aptamers called SOMAmers (slow off-rate modified aptamers) containing modified nucleotides with functional groups absent in natural DNA (21, 30). In addition to the polar and charge-charge contacts typical of conventional aptamer-target interactions, these novel base modifications mediate hydrophobic interactions between SOMAmers and their targets, leading to significant improvements in binding affinity and slower off-rates. The modified nucleotides also provide convenient handles for targeted post-SELEX modification of SOMAmers aimed at further improving their binding affinity, functional activity, and metabolic stability. We set out to identify SOMAmers that bind to human IL-6 with high affinity and specificity and inhibit the first and essential step in the IL-6-signaling pathway, binding of IL-6 to its cell surface receptors IL-6Rα and gp130. RNA and 2′fluoropyrimidine-modified aptamers to IL-6Rα have been recently reported, but none was inhibitory (51). Herein, we describe the discovery and characterization of two SOMAmers, each possessing a different hydrophobic modification. Both display high affinity binding to human IL-6 and neutralizing activity in functional cell-based assays but differ in species cross-reactivity. These SOMAmers have the potential to be effective inhibitors of IL-6-mediated signaling *in vivo*, offering an alternative treatment option for inflammatory diseases.

## EXPERIMENTAL PROCEDURES

### 

#### 

##### Proteins

Recombinant human IL-6 was purchased from PeproTech (Rocky Hill, NJ, catalog no. 200-06) for SELEX and binding assays and from R&D Systems (Minneapolis, MN, catalog no. 206-IL-050/CF) or EMD Millipore (Billerica, MA, catalog no. IL006) for the luciferase gene reporter assay. Recombinant rat IL-6 (catalog no. 506-RL-050/CF) and mouse IL-6 (catalog no. 406-ML-005/CF) were purchased from R&D Systems. Soluble human IL-6 receptor was purchased from Sigma (catalog no. I5771). Glycosylated human IL-6 was purchased from GenWay Biotech (San Diego, catalog no. 10-006-22054). Cynomolgus monkey IL-6 was prepared in-house as follows. The cynomolgus monkey IL-6 gene (*Macaca fascicularis,* GenBank^TM^ accession no. AB000554) with six repetitive histidine codons (CATCATCATCATCATCAT) was cloned into pcDNA5/FRT (Invitrogen, catalog no. V6010-20) and co-transfected with pOG-44 (Invitrogen, catalog no. V6005-20) into Flp-In^TM^ CHO cells (Invitrogen, catalog no. R758-07) to establish a stable cell line. Expressed monkey IL-6 was purified from supernatants of the cell culture using nickel-nitrilotriacetic acid His-Bind® resin and buffer kit (EMD Millipore, catalog nos. 0666 and 70899) according to the manufacturer's instructions. Protein concentration was determined by ELISA (R&D Systems, catalog no. D6050).

##### SOMAmer Synthesis

SOMAmers were prepared by solid phase synthesis using the phosphoramidite method (31) with some adjustments to the protocol to account for unique base modifications. Modified nucleoside phosphoramidite and triphosphate monomers were synthesized according to protocols described previously (30, 32). Biotin was added to SL1032 as a biotin serinol phosphoramidite and to SL1025 as a photo-cleavable biotin phosphoramidite, along with a Cy3 phosphoramidite. All phosphoramidites were purchased from Glen Research, Sterling, VA. SOMAmers with 5′-PEG modifications were prepared via PEG-NHS ester conjugation to hexylamine-modified SOMAmers using standard methods.

##### SOMAmer Discovery

SOMAmers were discovered using the SELEX process described in Gold *et al.* (21), from a modified DNA library with 40 random positions containing either 5-(*N*-benzylcarboxamide)-2′-deoxyuridine (Bn-dU) or 5-[*N*-(1-naphthylmethyl)carboxamide]-2′-deoxyuridine (Nap-dU) in place of dT. The 40 random positions (*N*_40_) were flanked by PCR priming regions with the following sequence: 5′-GATGTGAGTGTGTGACGAG*N*_40_CACAGAGAAGAAACAAGACC-3′. Recombinant human IL-6 was biotinylated by covalent coupling of NHS-PEO4-biotin (Thermo Scientific, Pittsburgh, PA, catalog no. 21329) to lysine residues according to the manufacturer's protocol. Protein (300 pmol in 50 μl) was exchanged into SB17T buffer (40 mm HEPES, pH 7.5, 102 mm NaCl, 5 mm KCl, 5 mm MgCl_2_, 1 mm EDTA, 0.05% Tween 20) with a Sephadex G-25 MicroSpin column. NHS-PEO4-biotin was added to 30 μm, and the reaction was incubated at 4 °C for 16 h. Unreacted NHS-PEO4-biotin was removed with a Sephadex G-25 MicroSpin column. Biotinylated IL-6 was equilibrated with a DNA library in SB17T, and complexes were captured via target protein biotins using MyOne-streptavidin paramagnetic beads (Invitrogen, catalog no. 65001). A kinetic challenge was applied to preferentially select sequences with slow complex dissociation rates. This was accomplished in rounds 2–5 by diluting the pre-equilibrated protein-SOMAmer complexes 20-fold in SB17T 15 min prior to capture, in rounds 6–7 by diluting 400-fold in SB17T 60 min prior to capture, and in round 8 by diluting 400-fold in SB17T containing 10 mm dextran sulfate 60 min prior to capture. After eight rounds of the SELEX process, the converged pools were cloned and sequenced.

##### Pool Sequencing and Analysis

Sequences for 48 clones from the enriched Bn-dU and Nap-dU pools were obtained using the Sanger method and analyzed using custom software that determines sequence counts/copy number and identifies common convergence patterns using a local alignment algorithm. Sixteen of these clones bound the MyOne-streptavidin beads in the absence of IL-6 protein, indicating they were streptavidin binders. Of the remaining clones, sequences with the highest representation (copy number) in the pool and sequences that shared common binding motifs were chosen for affinity screening. SOMAmers and their truncated variants were prepared synthetically as described previously (32).

##### Determination of Consensus Sequence

The enriched Bn-dU and Nap-dU pools were also sequenced using 454 pyrosequencing technology to acquire a larger number of sequences. For each pool, the DNA was amplified with 454 primers, and the PCR product was purified and normalized using a Sequa Prep normalization plate (Invitrogen, catalog no. A10510-01). The eluate was run on a gel to confirm the size and purity of each amplicon. The purified PCR product was sequenced at the 454 pyrosequencing facility at the University of Colorado Health Science Center (Aurora, CO). 14,404 Bn-dU and 7,758 Nap-dU sequences were acquired and analyzed using the algorithm described above.

##### Solution Measurement of Equilibrium Binding Constants (K_d_)

Equilibrium binding constants of SOMAmers were measured in SB17T at 37 °C as described by Gold *et al.* (21). Briefly, radiolabeled SOMAmer was equilibrated with various concentrations of IL-6 protein, and IL-6-SOMAmer complexes were captured with ZORBAX PSM-300 resin (Agilent Technologies, Santa Clara, CA) and quantified with a phosphorimager. The fraction of SOMAmer captured was plotted as a function of IL-6 concentration, and data were fit to a three-parameter sigmoid dose-response model to determine the *K_d_* value.

##### Surface Plasmon Resonance (SPR) Measurement of Interaction Kinetics

Kinetic analysis of SOMAmer binding to IL-6 was performed using a 404pi biosensor (BiOptix, Boulder, CO). Biotin-labeled SOMAmer was immobilized on a streptavidin-coated sensor surface by injection of a 300 nm solution in running buffer (SB17T) for 17.5 min at a flow rate of 20 μl/min. Binding was initiated by injection of recombinant human IL-6 in running buffer for 3.5 min at 100 μl/min (association phase), followed by injection of running buffer alone for 60 min at 100 μl/min (dissociation phase). Data were collected at 0, 4, 8, 16, 32, and 64 nm IL-6 with regeneration between runs using 10 mm NaOH. All data were collected at 37 °C, and each curve was referenced to a paired streptavidin-coated surface without SOMAmer. Sensorgrams were generated by plotting response units as a function of time for each IL-6 concentration after subtraction of control data without IL-6. Sensorgram data for all IL-6 concentrations were globally fit to determine the binding model parameters, namely the association rate constant (*k*_on_), the dissociation rate constant (*k*_off_), and the maximum SPR response (*R*_max_). Model input includes total protein concentration (*P_t_*), response units as a function of time (*R*(*t*)), and the time when the dissociation phase begins (*t_d_*). If a reasonable three-parameter fit (*k*_off, 1_, *k*_on, 1,_ and *R*_max, 1_) was not achieved with a one-site binding model (*n* = 1), a two-site binding model (*n* = 2) was applied using a six-parameter fit (*k*_off, 1_, *k*_on, 1_, and *R*_max, 1_, and *k*_off, 2_, *k*_on, 2_, and *R*_max, 2_). Equations 1–5 that govern the one- and two-site binding models are as follows:

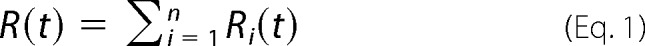
 for *t* ≤ *t_d_*

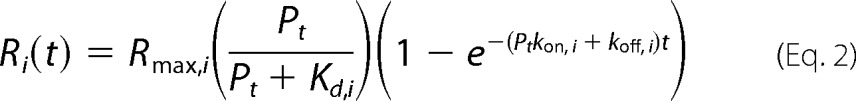
 where

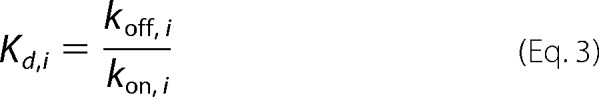
 for *t* > *t_d_*

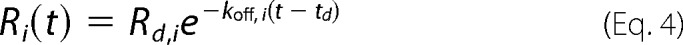
 where

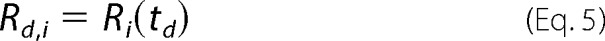
 and *R_i_*(*t_d_*) is defined by Equation 2.

##### Luciferase Gene Reporter Assay

The STAT-responsive sequence (5′-TGTTGCTCAATCGACTTCCCAAGAACAGGCTGTTGCTCAATCGACTTCCCAAGAACAGGCTGTTGCTCAATCGACTTCCCAAGAACAGGCTGTTGCTCAATCGACTTCCCAAGAACAG-3′), which contains the STAT consensus sequence 5′-TT(*N*_4–6_)AA-3′, was cloned into the pGL3-promoter vector (Promega, Madison, WI, catalog no. E1761) (33). This construct and pWL-neo plasmid (Stratagene, La Jolla, CA, catalog no. 200285-85) were co-transfected into HeLa cells, and a stable cell line was established (L4 cells).

L4 cells were plated in DMEM containing 10% FBS at 5 × 10^4^ cells per well in a 96-well white plate (Corning Glass, Corning, NY, catalog no. 3903) and cultured for 1 day at 37 °C in a CO_2_ incubator. Recombinant human IL-6 (10 ng/ml) was incubated with or without IL-6 SOMAmer and was added into supernatants. Cells were cultured for 1 day at 37 °C in a CO_2_ incubator. After culture, luciferase substrate reagent (TOYO B-Net, Tokyo, Japan, catalog no. 302-16163) was added to the cells for 30 min at ambient temperature after discarding supernatants. Luminescence was measured with a Wallac 1420 ARVO Light (PerkinElmer Life Sciences).

##### Cell Proliferation Assays

U266B1 cells (human myeloma, ATCC catalog no. TIB-196) were suspended with SOMAmer (1, 10, or 100 μg/ml) or tocilizumab (ACTEMRA 200 mg, Genentech, Inc., South San Francisco, CA; 1, 10, or 100 μg/ml) in RPMI 1640 medium containing 10% FBS at 10^4^ cells per well and cultured for 30 min at 37 °C in a 5% CO_2_ incubator. Human recombinant IL-6 (R&D Systems, catalog no. 206-IL; 100 ng/ml) was added, and cells were incubated for 2 days at 37 °C. AlamarBlue (Bio-Rad, catalog no. BUF012A) was added, and cells were incubated an additional 2–3 h at 37 °C. Fluorescence (excitation at 560 nm and emission at 590 nm) was measured with a luminometer (Wallac 1420 ARVO Light, PerkinElmer Life Sciences).

HepG2 cells (human hepatoma, ECACC catalog no. EC85011430) or U87MG cells (human glioma, ECACC catalog no. EC89081402) were plated in DMEM containing 10% FBS at 10^4^ cells per well in a 96-well plate and cultured for 1 day at 37 °C in a 5% CO_2_ incubator. SOMAmer (0.83 or 8.3 μm) or tocilizumab (1 or 10 μm) was added, and cells were incubated for 7 days at 37 °C. Proliferation was measured with AlamarBlue as described above.

##### Serum Stability Assay

SOMAmer (0.5 μm) was incubated with 90% human serum (Innovative Research, Novi, MI, catalog no. IPLA-SER) in SB17T buffer containing 0.01% Tween 20. Samples were incubated at 37 °C, and aliquots were drawn at various time points from 0 to 48 h. A control DNA of different lengths was added to each sample for normalization. Aliquots were extracted once with phenol and once with chloroform and concentrated with a YM-10 molecular weight cutoff filter (EMD Millipore, catalog no. MRCPRT010). Samples were analyzed by denaturing PAGE using a 10% polyacrylamide/urea gel, and SOMAmer was stained with SYBR Gold nucleic acid gel stain (Invitrogen, catalog no. S-11494). Stained DNA was imaged with a FluorChem Q fluorescent image analyzer (Alpha Innotech, San Leandro, CA) and quantified using the AlphaView Q software package. The fraction of intact SOMAmers was normalized to the control DNA sample at each time point.

##### IL-6 Receptor Binding Assay

Soluble IL-6Rα expressed in Sf21 insect cells was coupled to the surface of a microtiter plate (Nunc, Roslilde, Denmark, catalog no. 468667) by passive adsorption. Biotinylated IL-6 (50 ng/ml) was mixed with different concentrations of SOMAmer in assay buffer (PBS with 1% BSA, 0.05% Tween 20, 5 mm MgCl*_2_*) and added to the plate. After incubating for 120 min at 25 °C with shaking at 200 rpm, unbound IL-6 was removed by washing with PBST Buffer (PBS with 0.05% Tween 20), and the amount of remaining biotinylated IL-6 was measured with streptavidin horseradish peroxidase (Thermo Scientific, catalog no. 21130) according to standard procedures. The percent of biotinylated IL-6 bound to soluble IL-6Rα (relative to the no-competitor control) was plotted as a function of SOMAmer concentration.

## RESULTS

### 

#### 

##### Discovery of High Affinity IL-6 SOMAmers

**FIGURE 1.**
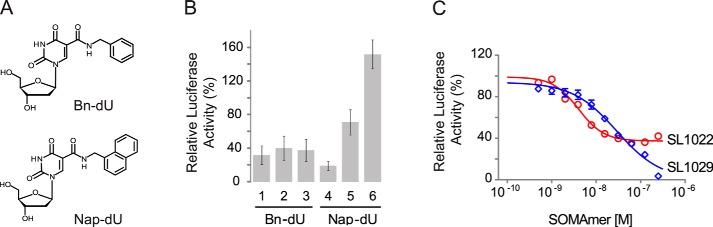
**Properties of SOMAmers discovered by the SELEX process.** *A,* structure of Bn-dU and Nap-dU. *B,* IL-6 inhibition activity of six high affinity SOMAmers (three Bn-dU and three Nap-dU) in the luciferase gene reporter assay. Percent luciferase activity was plotted relative to a no-SOMAmer control sample. Each *bar* represents the mean ± S.E. with two replicates in three independent experiments. *C,* dose-dependent inhibition of IL-6 by lead SOMAmers SL1022 and SL1029 in the luciferase gene reporter assay. Percent IL-6 activity (relative to a no-SOMAmer control sample) was plotted as a function of SOMAmer concentration. Each data point represents the mean ± S.E. with four replicates for each condition. The data were fit to a four-parameter sigmoid dose-response model, and IC_50_ values were determined. The reported IC_50_ value for SL1022 is an apparent IC_50_ because incomplete inhibition (<100%) was observed at the highest SOMAmer concentration tested.

IL-6 SOMAmers were isolated using the SELEX process described by Gold *et al.* (21) with modified DNA libraries containing 40 random positions and either Bn-dU or Nap-dU replacing dT (Fig. 1*A*). Selections included a kinetic challenge (addition of a large molar excess of the nonspecific competitor dextran sulfate after equilibration of IL-6 and the DNA library) to favor sequences with slow dissociation rates. Following eight rounds of selection and amplification, affinity-enriched libraries were sequenced, and three of the most abundant sequences from each enriched library representing different sequence patterns were synthesized and screened for IL-6 binding. High affinity binding to human recombinant IL-6 protein was observed for all six SOMAmers, with *K_d_* values ranging from 1 to 5 nm (data not shown).

##### IL-6 SOMAmers Act as Antagonists in Cell Assays

The six SOMAmers described above were evaluated for their ability to inhibit IL-6-mediated activation of cellular responses using a bioluminescent gene reporter assay. Treatment of L4 cells with IL-6 resulted in an IL-6-dependent increase in luciferase activity. Percent luciferase activity in the presence of SOMAmer (relative to the control with no SOMAmer) is plotted in Fig. 1*B*. All three of the Bn-dU and two of the three Nap-dU SOMAmers inhibited IL-6-mediated luciferase activity under these conditions. SOMAmer inhibition activity was confirmed in a cell proliferation assay (data not shown). The Bn-dU SOMAmer 3 (SL1022) and the Nap-dU SOMAmer 4 (SL1029) were chosen for further evaluation.

Dose-dependent inhibition of IL-6 by SL1022 and SL1029 was demonstrated in the luciferase gene reporter assay. Luciferase activity was plotted as a function of SOMAmer concentration (Fig. 1*C*), and data were fit to a four-parameter sigmoidal dose-response model to determine half-maximal inhibitory concentration (IC_50_) values. The inhibition profiles for Bn-dU and Nap-dU SOMAmers were markedly different. Bn-dU SOMAmer SL1022 exhibited an apparent IC_50_ value of about 4 nm, but achieved only 60% inhibition at the highest SOMAmer concentration tested (256 nm), whereas Nap-dU SOMAmer SL1029 achieved nearly 100% inhibition, but with a higher IC_50_ value of about 30 nm. Additional optimization of SL1022 and SL1029 was initiated to further enhance inhibitory potency and achieve more complete inhibition of IL-6.

##### Deep Sequence Analysis and Consensus Identification

**FIGURE 2.**
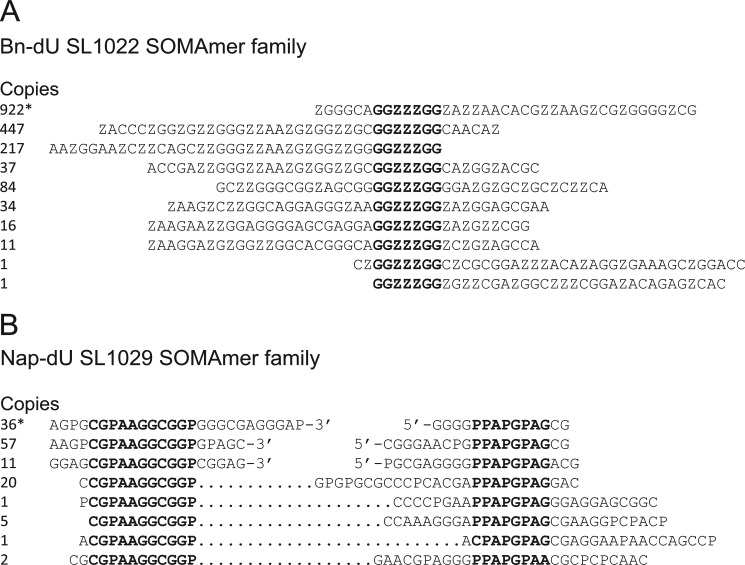
**Conserved sequence patterns in lead SOMAmers.** *A,* alignment of SL1022 (indicated with *) with additional sequences from the Bn-dU SELEX pool containing the consensus motif GG*ZZZ*GG (Z represents Bn-dU). The number of copies of each sequence in a pool of 14,404 is shown. *B,* alignment of SL1029 (indicated with *) with additional sequences from the Nap-dU SELEX pool containing the consensus motifs CG*P*AAGGCGG*P* and *PP*A*P*G*P*AG (P represents Nap-dU). The number of copies of each sequence in a pool of 7,758 is shown.

The two lead SOMAmers, SL1022 and SL1029, were initially identified from an analysis of 48 sequences from their respective affinity-enriched pools. A larger number of sequences was acquired by re-sequencing the Bn-dU and Nap-dU pools using Next Generation Sequencing technology. Pattern search analysis of 14,404 Bn-dU and 7,758 Nap-dU sequences led to the identification of consensus motifs in SL1022 and SL1029 within a family of related sequences that may be required for SOMAmer binding to IL-6 (Fig. 2). Bn-dU SOMAmer SL1022 was the most abundant sequence in the Bn-dU pool (922 copies) and contained the consensus motif GG*ZZZ*GG (*Z* represents Bn-dU), whereas Nap-dU SOMAmer SL1029 contained two circularly permuted consensus motifs, CG*P*AAGGCGG*P* and *PP*A*P*G*P*AG (*P* represents Nap-dU), with intervening sequence of variable length.

##### SOMAmer Truncation

**FIGURE 3.**
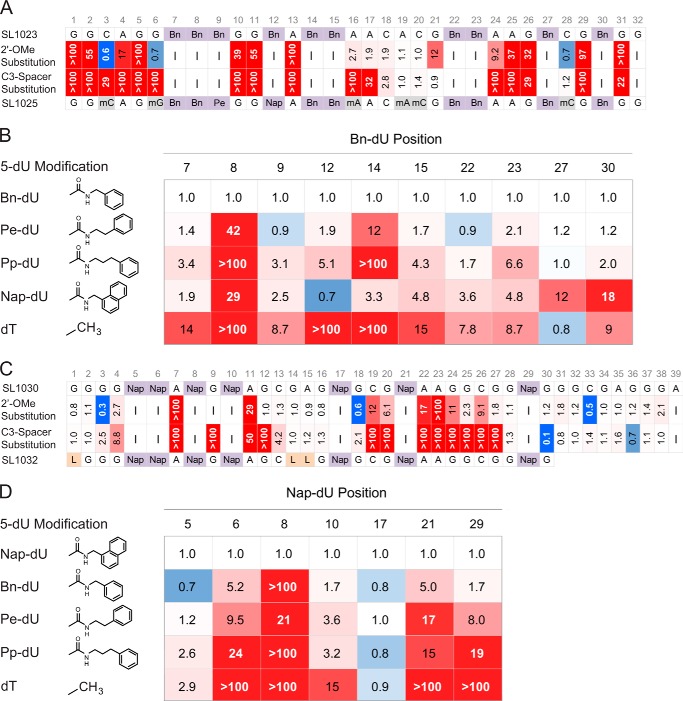
**Post-SELEX optimization of truncated IL-6 SOMAmers SL1023 and SL1030.** For all panels, affinity ratios (*K*_*d*_^variant^/*K*_*d*_^parent^) are reported and *shaded* with a color gradient from *blue* (affinity enhancement) to *red* (affinity loss), where the color intensity represents the magnitude of the affinity ratio. No affinity data were acquired at positions marked with -. *A,* 2′-OMe and C3-spacer substitution scans at A, C, and G positions of SL1023. The sequence of lead optimized truncate SL1025 is shown with modifications indicated (*Bn,* Bn-dU; *Pe,* Pe-dU; *Nap,* Nap-dU; *mC,* 2′-OMe C; *mG,* 2′-OMe G; *mA,* 2′-OMe A). *B,* 5-position modification scans at Bn-dU positions of SL1023. Structures of hydrophobic modifications Bn-dU, Pe-dU, Pp-dU, and Nap-dU are illustrated. *C,* 2′-OMe and C3-spacer substitution scans at A, C, and G positions of SL1030. The sequence of lead optimized truncate SL1032 is shown with modifications indicated (Nap, Nap-dU; *L*, C3-spacer). *D,* 5-position modification scans at Nap-dU positions of SL1030.

The minimal binding domains of SL1022 and SL1029 were determined empirically by synthesizing variants with 5′- and 3′-truncations and screening for binding activity (data not shown). This iterative process resulted in the reduction of Bn-dU SOMAmer SL1022 to a 32-nucleotide sequence (SL1023) with equivalent affinity as its full-length parent SOMAmer (Fig. 3*A*). Truncation of the Nap-dU SOMAmer SL1029 led to a 39-nucleotide sequence (SL1030) (Fig. 3*C*) and to a 30-nucleotide sequence (SL1031) after further rounds of optimization. A list of all SOMAmers described in this report can be found in Table 1.

**TABLE 1:** **Key to SOMAmers described in this report** Modifications are denoted as follows: Bn (Bn-dU), Nap (Nap-dU), Pe (Pe-dU), 2′-OMe (2′-methoxy), L (C3-spacer), OH (hydroxyl), PEG (polyethylene glycol, 40 kDa).

SOMAmer	Version	Length	Composition	5′-Terminal group
		*nt*		
SL1022	Parent	78	Bn	OH
SL1023	Truncate	32	Bn	OH
SL1023dT	Unmodified truncate	32	dT	OH
SL1024	Optimized truncate	32	Bn, 2′-OMe	OH
SL1025	Lead optimized truncate	32	Bn, 2′-OMe, Pe, Nap	OH
SL1026	Lead optimized truncate	32	Bn, 2′-OMe, Pe, Nap	PEG
SL1027	Optimized truncate	32	Bn, 2′-OMe, Pe, Nap	OH
SL1029	Parent	79	Nap	OH
SL1030	Truncate	39	Nap	OH
SL1031	Truncate	30	Nap	OH
SL1031dT	Unmodified truncate	30	dT	OH
SL1032	Lead optimized truncate	30	Nap, L	OH
SL1033	Lead optimized truncate	30	Nap, L	PEG

##### Post-SELEX SOMAmer Optimization

The affinity, specificity, and nuclease stability of aptamers can be improved significantly with modifications to the phosphodiester backbone, ribose or deoxyribose sugar, and base components (28). Modification of the 2′-sugar position with a methoxy group (2′-OMe) provides very effective resistance to DNase and RNase activity *in vivo*, but the added methoxy groups have the potential to interfere with target protein binding. To assess the tolerance of SOMAmers SL1023 and SL1030 to 2′-OMe substitutions, each dA, dC, and dG nucleotide was substituted individually with the corresponding 2′-OMe derivative, and the affinities of the 21 synthetic variants of SL1023 and 31 variants of SL1030 were measured. A similar scan was performed with a C3-spacer (3-carbon alkyl linker in place of the nucleoside while preserving the spacing) to determine the contribution of specific nucleosides to SOMAmer binding. Results are illustrated in Fig. 3. Affinity ratios (*K*_*d*_^variant^/*K*_*d*_^parent^) are reported and shaded with a color gradient from *blue* (affinity enhancement) to *red* (affinity loss) (Fig. 3), where the color intensity represents the magnitude of the affinity ratio.

Both 2′-OMe and C3-spacer substitutions had no effect on the binding affinity of Bn-dU SOMAmer SL1023 in the region of A16–C20 and C18–G21, respectively, suggesting that this region of the SOMAmer is not involved in IL-6 binding (Fig. 3*A*). A slight enhancement in binding affinity was observed with 2′-OMe substitutions at positions C3, G6, and C28, and an additional C3-spacer substitution was tolerated at C28. A significant (>5-fold) affinity loss was observed with a 2′-OMe or a C3-spacer substitution at any of the other positions, indicating their role in maintaining the SOMAmer structure for IL-6 binding.

The interaction surface between SL1023 and IL-6 was probed using alternative hydrophobic dU modifications at Bn-dU positions to identify contact points and opportunities for affinity enhancement. Forty singly substituted variants of SL1023 were synthesized with each Bn-dU replaced by 5-[*N*-(phenyl-2-ethyl)carboxamide]-2′-deoxyuridine (Pe-dU, possessing one additional alkyl carbon in the linker), 5-[*N*-(phenyl-3-propyl)carboxamide]-2′-deoxyuridine (Pp-dU, possessing two additional alkyl carbons in the linker), Nap-dU, or dT. Variants were tested for binding, and the results are summarized in Fig. 3*B*. Substitution of Bn-dU at position 8 with any other dU modification resulted in loss of binding to IL-6, suggesting that this residue plays a critical role in the SOMAmer interaction with IL-6. Bn-dU at position 14 in SL1023 tolerated an additional benzyl ring but not increased linker length, whereas at other Bn-dU positions (27 and 30), changing the linker length had no deleterious effect, but an additional ring was not tolerated. Substitutions with any dU modification (Nap-dU, Pe-dU, and Pp-dU) were tolerated at Bn-dU positions 7, 9, 12, 15, 22, and 23. All but one Bn-dU (at position 27) were sensitive to dT replacement, with complete loss of affinity observed from substitutions at positions 8, 12, or 14, and partial loss of affinity with substitutions at positions 7, 9, 15, 22, or 23. All the binding data for these substitutions were supported by corresponding differences in inhibitory potency, as measured in the luciferase gene reporter assay (data not shown).

A similar series of substitution scans was done with the Nap-dU SOMAmer SL1030. Single 2′-OMe substitutions had no deleterious effect between G12 and G16, and C3-spacer substitutions were tolerated between C13 and G16 (Fig. 3*C*). This region is between the two consensus motifs of the Nap-dU SOMAmer. Both 2′-OMe and C3-spacer substitutions were tolerated at nearly all positions within the 3′ end of the SOMAmer suggesting that this region is not required for interaction with the IL-6 protein. Consistent with these observations, we were able to remove positions 31–39 in a subsequent round of truncation variants without compromising the activity of the Nap-dU SOMAmer. Almost all the residues forming the conserved motifs did not tolerate any substitutions. Affinity was significantly reduced by dT substitutions at all Nap-dU positions except 5 and 17. Of the seven Nap-dU nucleotides present in SL1030, position 17 is the only one that is not part of a consensus motif (Fig. 3*D*). This implies that hydrophobic modifications at all other positions contribute to the SOMAmer structure and/or interaction with IL-6.

**FIGURE 4.**
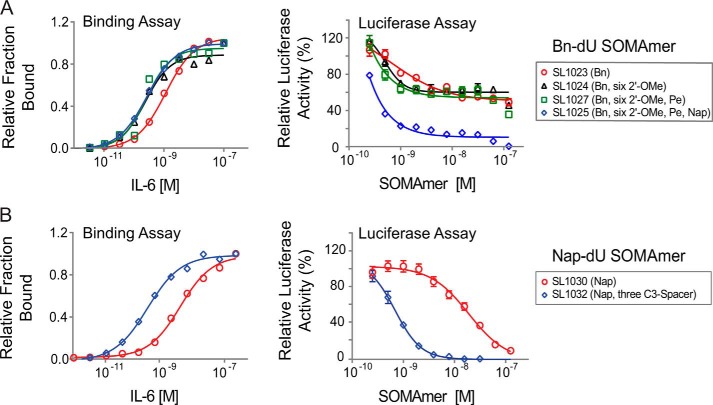
**Improvements in IL-6 binding and inhibition activity after SOMAmer optimization.** Dose-dependent binding and inhibition of IL-6 by Bn-dU SOMAmers (*A*) and Nap-dU SOMAmers (*B*) and their optimized variants. Equilibrium binding constants of truncated SOMAmers SL1023 and SL1030 and optimized variants were measured using a solution affinity assay. The fraction of complexed SOMAmer was plotted as a function of IL-6 concentration, and equilibrium binding constants (*K_d_*) were determined by fitting the data to a four-parameter sigmoid dose-response model. Functional inhibitory activity (IC_50_) values of truncated SOMAmers SL1023, SL1030, and optimized variants were measured in the luciferase gene reporter assay as described in Fig. 1*C*. Each data point represents the mean ± S.E. with four replicates for each condition.

From the single substitution data summarized in Fig. 3, more than 100 combinations of tolerated 2′-OMe and C3-spacer substitutions and beneficial 5-dU substitutions were synthesized for each SOMAmer and evaluated for IL-6 binding and inhibition activity (data not shown). Many combinations with up to a 10-fold affinity improvement were identified for both the Bn-dU and Nap-dU SOMAmers. The most favorable combination of substitutions in the Bn-dU SOMAmer was observed in variant SL1025, which contained six 2′-OMe groups (at positions C3, G6, A16, A19, C20, and C28) and two 5-dU mutations (Bn-dU9 → Pe-dU9 and Bn-dU12 → Nap-dU12). The affinity of SL1025 for IL-6 (*K_d_* = 0.2 nm) was ∼5-fold greater than that of its precursor SL1023 (*K_d_* = 1 nm). The Nap-dU SOMAmer tolerated fewer substitutions; however, compared with its precursor SL1030, a 10-fold affinity improvement was observed for variant SL1032 (*K_d_* = 0.2 nm), with three C3-spacer substitutions at positions G1, G14, and A15, and the 3′-terminal nine nucleotides removed (see Fig. 4). These two optimized variants were chosen as leads for further analysis in functional activity assays.

##### Single Bn-dU to Nap-dU in SOMAmer SL1025 Leads to Complete Inhibition of IL-6

The inhibitory activities of the lead Bn-dU and Nap-dU SOMAmers were compared in the luciferase gene reporter assay before and after optimization. Optimized Nap-dU SOMAmer SL1032 (IC_50_ = 0.9 nm) was ∼30-fold more potent than its truncated un-optimized parent SL1030 (IC_50_ = 30 nm) and its full-length un-optimized parent SL1029 (Figs. 4*B* and 1*C*). Optimized Bn-dU SOMAmer SL1025 also increased in potency compared with its full-length un-optimized parent SL1022, as evident from the 20-fold shift in IC_50_ (0.2 nm *versus* 4 nm) (Figs. 4*A* and 1*C*). Surprisingly, inhibition of IL-6 by SL1023 increased from 60% to nearly 100% after optimization to SL1025. This observed increase was attributable to the single substitution of Bn-dU to Nap-dU at position 12. A direct comparison of inhibition dose-response curves between SL1025 (with Nap-dU12) and SL1027 (SL1025 with Bn-dU12) revealed that this single substitution was responsible for the observed increase in percent inhibition (Fig. 4*A*). It is important to note that the IC_50_ values are limited by the concentration of IL-6 used in this assay (0.5 nm), and therefore, the true inhibitory potency of the lead SOMAmers may be greater than those reflected in the IC_50_ values. The combined effects of SOMAmer truncation and optimization on IL-6 binding and inhibition are summarized in Table 2.

**TABLE 2:** **Properties of SOMAmers before and after post-SELEX optimization** 95% confidence intervals of curve fit values are reported in parentheses after *K_d_*, IC_50_ and Percent Inhibition values.

SOMAmer	Modification	Version	Length	*K_d_*	IC_50_ (nM)	Percent inhibition*^a^*
			*nt*	*nm*	nm	
SL1022	Bn-dU	Parent	78	3.4 (2.0–4.9)	4.0 (2.8–5.7)*^b^*	62 (57–68)
SL1025	Bn-dU	Lead optimized truncate	32	0.16 (0.11–0.20)	0.20 (0.15–0.27)	90 (81–102)
SL1029	Nap-dU	Parent	79	2.1 (1.9–2.3)	30 (17–77)	100 (78–106)
SL1032	Nap-dU	Lead optimized truncate	30	0.19 (0.17–0.22)	0.87 (0.78–0.98)	102 (101–103)

*^a^* Percent inhibition observed at highest SOMAmer concentration tested.

*^b^* Apparent IC_50_ is shown.

##### Kinetic Analysis of SOMAmer Binding to IL-6

**FIGURE 5.**
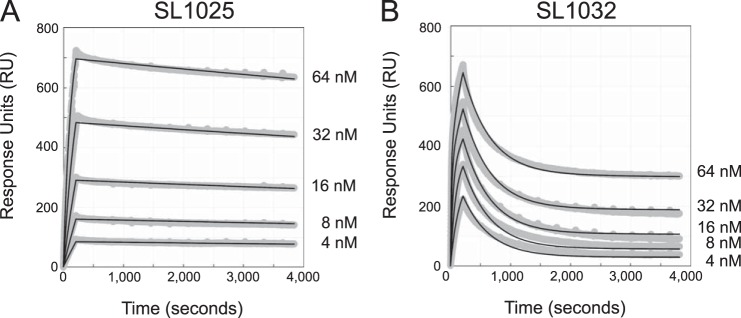
**Kinetic evaluation of SL1025 and SL1032 binding to IL-6.** Kinetic constants (*k*_on_ and *k*_off_) for the interaction between IL-6 and SOMAmer were determined by SPR analysis with a BiOptix 404pi biosensor. Raw data are shown in *gray* with curve fits overlaid in *black* and IL-6 concentrations indicated to the *right* of each curve. *A,* SL1025 data were fit globally to a one-site binding model to determine kinetic constants. *B,* SL1032 data were fit globally to a two-site binding model to determine kinetic constants.

A kinetic evaluation of SL1025 and SL1032 binding to IL-6 was performed at 37 °C using SPR. Biotin-labeled SOMAmer was immobilized on a streptavidin-coated surface, and IL-6 was injected for 3.5 min (association phase), followed by buffer without IL-6 for 60 min (dissociation phase). Response units were plotted as a function of time for all five IL-6 concentrations (Fig. 5). A global fit of the SL1025 data was performed with a one-site binding model, and association and dissociation rate constants were determined (*k*_on_ = 1.2 × 10^5^ m^−1^ s^−1^ and *k*_off_ = 2.8 × 10^−5^ s^−1^). The equilibrium binding constant calculated as the ratio of *k*_off_/*k*_on_ (*K_d_* = 2.3 × 10^−10^ m) was consistent with solution measurements. Rate constants for SL1032 were determined in a similar manner using a two-site binding model (*k*_on, 1_ = 7.9 × 10^4^ m^−1^ s^−1^, *k*_off, 1_ = 6.9 × 10^−6^ s^−1^, *k*_on, 2_ = 1.4 × 10^6^ m^−1^ s^−1^, and *k*_off, 2_ = 2.2 × 10^−3^ s^−1^). The equilibrium binding constant for the high affinity ligand interaction (*K_d_*_,1_ = *k*_off, 1_/*k*_on, 1_ = 8.7 × 10^−11^ m) was also consistent with solution measurements.

##### Cross-species Reactivity and Effect of Protein Glycosylation on SOMAmer Activity

**FIGURE 6.**
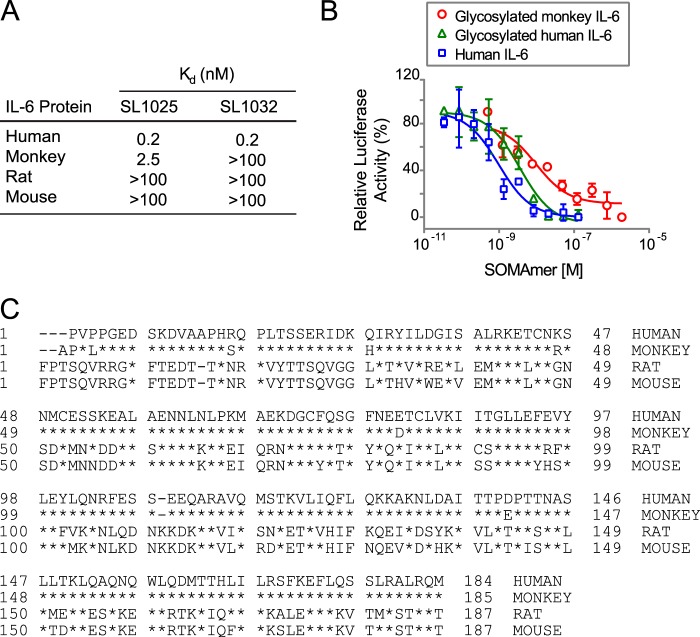
**Cross-reactivity of SOMAmers with IL-6 from different species.** *A,* equilibrium binding constants (*K_d_*) of SL1025 and SL1032 to human, cynomolgus monkey, rat, and mouse IL-6 protein. *K_d_* values were determined as described in Fig. 4*A. B,* dose-dependent inhibition of human, glycosylated human, and glycosylated cynomolgus monkey IL-6 by SL1026 in the luciferase gene reporter assay. IC_50_ values were determined as described in Fig. 1*C. C,* amino acid sequence alignment of mature human, cynomolgus monkey, rat, and mouse IL-6 proteins. Sequences of IL-6 proteins with Swiss-Prot accession numbers P05231 (human), P79341 (cynomolgus monkey), P20607 (rat), and P08505 (mouse) were aligned with UniProt after removing the signaling peptide sequence at the N terminus. Amino acid sequences are presented in single letter code, and residues that are identical with the sequence of human IL-6 protein are indicated with a *star*.

The binding properties of SL1025 and SL1032 to IL-6 from different species, including rat, mouse, and monkey, were profiled. Neither SOMAmer showed measurable binding to any of these orthologs, with the exception of SL1025, which bound monkey IL-6, although with a 10-fold reduction in affinity (*K_d_* = 2.5 nm) (Fig. 6*A*). The monkey IL-6 used for this study was expressed in eukaryotic cells and was therefore glycosylated, whereas the human IL-6 used for SELEX was expressed in *Escherichia coli* and was nonglycosylated. The effect of target glycosylation on SL1025 activity was determined by comparing inhibition of glycosylated and nonglycosylated human IL-6 in the gene reporter assay along with glycosylated monkey IL-6 (Fig. 6*B*). SL1025 inhibited all three forms of IL-6, but a reduction in potency (4-fold) was observed with glycosylated human IL-6, and a further reduction (3-fold) was observed with glycosylated monkey IL-6.

##### Modified Nucleotides Impart Resistance to Nuclease Attack

**FIGURE 7.**
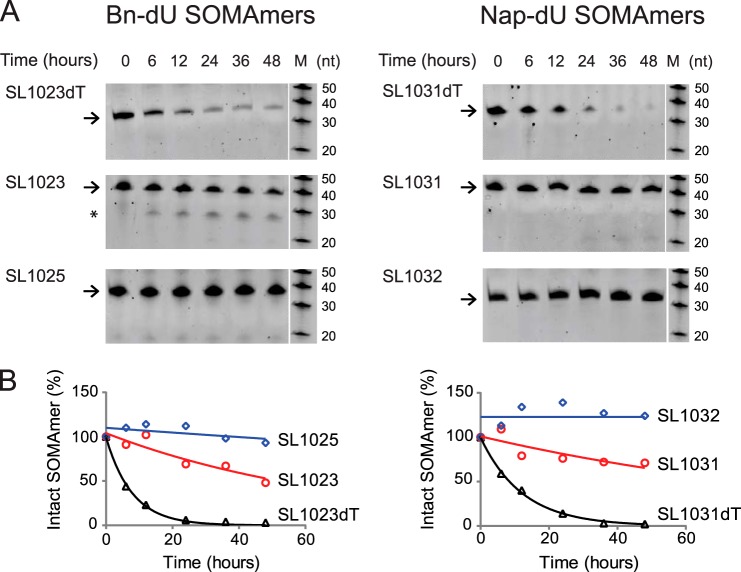
**Stability of SOMAmers in human serum *in vitro*.** *A,* gel images of truncated SOMAmers SL1023 and SL1031, dT controls SL1023dT and SL1031dT, and optimized SOMAmers SL1025 and SL1032 after serum exposure for the indicated amount of time. DNA markers (*M*) were run on a different part of the gel. Intact SOMAmers are indicated with *arrows*, and a stable metabolite of SL1023 is marked with an *asterisk. B,* graphical representation of PAGE results. The fraction of intact SOMAmer (relative to the zero time point control sample) was plotted as a function of time. The data were fit to a one-phase exponential decay model to determine half-life values.

The sensitivity of optimized SOMAmers SL1025 and SL1032 to serum nucleases was measured in an *in vitro* nuclease stability assay. Active SOMAmers were compared with un-optimized versions SL1023 and SL1031 and inactive analogs, in which all modified dU residues were replaced with natural dT residues (SL1023dT and SL1031dT). All SOMAmers tested contained a 3′-inverted dT (3′-idT) group to block 3′- to 5′-exonuclease activity (34). SOMAmers were incubated with 90% human serum at 37 °C for up to 48 h, and samples were analyzed at different time points by denaturing PAGE (Fig. 7*A*). Percent intact SOMAmer was plotted as a function of time and fit to a one-phase exponential decay model to determine half-life (Fig. 7*B*). As expected, the dT controls were rapidly cleaved (*t*_½_ = 5.5 h for SL1023dT and *t*_½_ = 8.5 h for SL1031dT). The un-optimized SOMAmers, however, were significantly more stable (*t*_½_ = 50 h for SL1023 and *t*_½_ = 77 h for SL1031). Nevertheless, some degradation of SL1023 and SL1031 was observed (along with a discrete metabolite of SL1023), indicating certain positions within the SOMAmers remained sensitive to nuclease cleavage. Further stability enhancement was achieved with the addition of 2′-OMe and C3-spacer substitutions, as very little cleavage occurred in 48 h with the optimized SOMAmers SL1025 and SL1032.

##### Comparison of IL-6 SOMAmer Inhibition Activity with Tocilizumab

**FIGURE 8.**
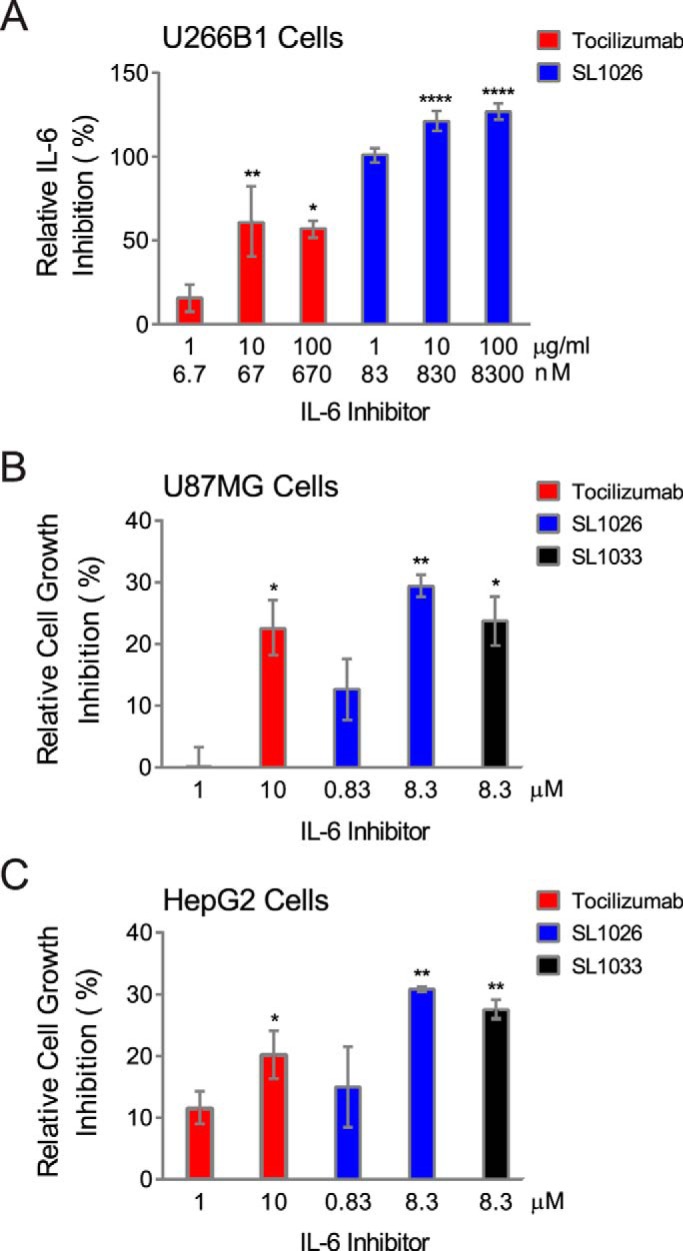
**Inhibition of tumor cell proliferation by SL1026, SL1033, and tocilizumab.** *A,* inhibition of U266B1 (human myeloma) cell proliferation. Cell proliferation was measured with AlamarBlue, and percent IL-6 inhibition (relative to a no-inhibitor control sample) was plotted. Each *bar* represents the mean ± S.E. of three experiments. The data were analyzed by one-way analysis of variance (Dunnett's two-tailed), and each data point was compared with control data without IL-6 inhibitor. Statistically significant differences are denoted as *, *p* value < 0.05; **, *p* value < 0.01; ****, *p* value < 0.0001. Inhibition of U87MG (human glioma) and HepG2 (human hepatoma) cell proliferation are illustrated in *B* and *C,* respectively. Percent inhibition of cell growth (relative to a no-inhibitor control sample) was plotted.

To prevent renal elimination in future *in vivo* studies, a branched 40-kDa PEG was conjugated to SOMAmers SL1025 and SL1032 to create SL1026 and SL1033, respectively (35, 36). Addition of the PEG moiety did not affect the inhibitory activity of the SOMAmers in the luciferase gene reporter assay (data not shown). We then compared the activity of Bn-dU SOMAmer SL1026 with tocilizumab in the U266B1 human myeloma cell proliferation assay as described under “Experimental Procedures.” SL1026 achieved complete inhibition of IL-6 at 1 μg/ml (83 nm), whereas tocilizumab achieved 60% inhibition at a roughly equivalent molar concentration (67 nm) (Fig. 8*A*). Inhibition of cell proliferation by SL1026 and SL1033 was also measured for two human tumor cell lines, HepG2 (hepatoma) and U87MG (glioma), and compared with tocilizumab. Percent proliferation was quantified relative to a no-inhibitor control and plotted in Fig. 8, *B* and *C*. Both SL1026 and SL1033 suppressed proliferation of U87MG and HepG2 cells to a greater extent than tocilizumab at similar molar concentrations.

##### SL1026 and SL1033 Block Binding of IL-6 to IL-6 Receptor

**FIGURE 9.**
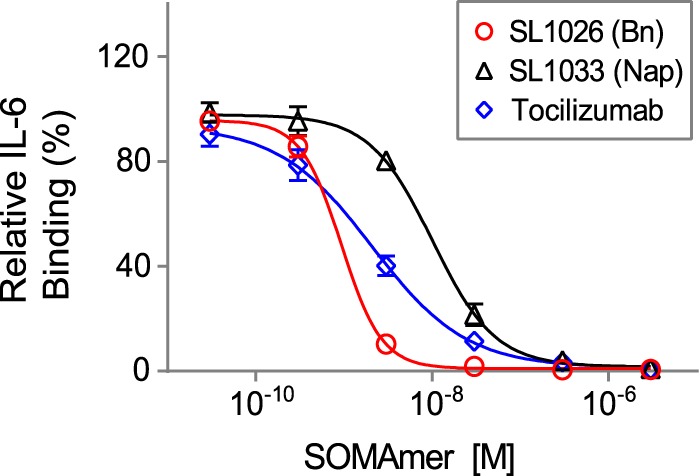
**SL1026 and SL1033 compete with soluble IL-6 receptor for IL-6 binding.** IL-6 binding to immobilized IL-6Rα was measured in the presence of SOMAmer or tocilizumab. Percent IL-6 binding (relative to a no-inhibitor control sample) was plotted as a function of inhibitor concentration. Each data point represents the mean ± S.E. with two replicates for each condition. The data were fit to a four-parameter sigmoid dose-response model.

To further understand the mechanism of inhibition, we developed a plate-based sandwich assay to determine whether SOMAmers block IL-6 binding to IL-6Rα, the first step in the IL-6 signaling pathway. We tested sIL-6Rα binding to biotinylated IL-6 preincubated with different concentrations of SOMAmer or tocilizumab. The percent of IL-6 bound to sIL-6Rα (relative to the no-competitor control) was plotted as a function of inhibitor concentration (Fig. 9). As the concentration of IL-6 inhibitor increased, the amount of bound IL-6 decreased, indicating that SL1026, SL1033, and tocilizumab block the binding of IL-6 to its receptor.

## DISCUSSION

Aptamers are an established technology for inhibiting protein function *in vitro* and *in vivo* (28, 29, 37). Aptamers bind their molecular targets with high affinity and specificity by virtue of surface shape and charge complementarities. SOMAmers are a new class of aptamers with enhanced functionality offering more favorable properties for target inhibition, including exquisite affinity (typical *K_d_* <1 nm) and slow dissociation rate (typical *t*_½_ >30 min). These properties are enabled by hydrophobic adducts at the 5-position of uridine that facilitate the formation of unique intramolecular structures and direct interactions with hydrophobic amino acids on the surface of the target protein (32). We performed the SELEX process against human IL-6 with two random libraries, one with benzyl modifications on the 5-position of dUTP (Bn-dU) and the other with naphthyl modifications (Nap-dU), and we identified two SOMAmers with similar binding affinity to IL-6 (*K_d_* = 2–3 nm) but different inhibitory properties *in vitro*. Optimization efforts resulted in improvements in affinity and nuclease stability, and more potent and complete inhibition of IL-6 activity.

Different sequence motifs were identified for SL1022 (Bn-dU) and SL1029 (Nap-dU). Although both modifications are planar, hydrophobic, and aromatic, and the SELEX process leading to the discovery of these two SOMAmers was identical, the size and chemical properties of the two modifications were sufficiently different to yield unique SOMAmer sequence solutions (and, by inference, structure solutions) for IL-6 binding. In other words, an apparently subtle change in the functional group of the side chain was sufficient to cause different sequences to be favored during affinity selections. This has been observed previously with modifications at the 2′-position of ribose; for example, SELEX aimed at the same target but accomplished with different starting libraries (including unmodified RNA, DNA, 2′-aminopyrimidine, and 2′-fluoropyrimidine RNA) has invariably led to completely different primary structure solutions to high affinity binding (38–40). Our results extend this observation to highly related modifications at the 5-position of uridine and provide further support for the notion that nucleic acid ligands represent precisely assembled structures in which individual nucleotides make aggregate contributions to scaffold assembly and presentation of key functional groups to their binding partner to enable the formation of high affinity complexes.

Post-SELEX optimization is commonly performed to shorten an aptamer and enhance affinity and nuclease resistance, which often leads to improved potency. For conventional aptamers, backbone protection has been necessary to improve the metabolic stability and achieve adequate plasma residence time for systemic use. RNA aptamers are typically selected with fluorine modifications on the 2′-ribose position of pyrimidines, and only purine positions require stabilization, most commonly with 2′-OMe. DNA SOMAmers are selected with no sugar modifications, and therefore all backbone positions are susceptible to endonuclease attack. Because addition of a methoxy group at any 2′-position might interfere with protein binding, we surveyed by chemical synthesis each dA, dG, and dC position of SL1023 individually to identify those that tolerated a 2′-OMe, and we screened combinations to find six positions in SL1025 that could be substituted without sacrificing binding activity. Unlike conventional RNA aptamers reported in the literature that are broadly substituted with 2′-OMe purines without loss of activity, a smaller number of 2′-OMe substitutions were tolerated in SL1025. This may be attributed in part to a conformational preference in DNA for the 2′-endo sugar pucker and its likely perturbation to the 3′-endo pucker upon 2′-OMe substitution. The 2′-endo conformation is energetically favorable in natural deoxyribose, whereas the 3′-endo conformation is preferred in ribose (RNA), 2′-OMe ribose, and 2′-fluororibose (41). The same preference for the 3′-endo conformation in the latter is likely responsible for the high degree of tolerance of the 2′-fluoropyrimidine, 2′-ribopurine nucleic acid libraries (which are often used in SELEX) toward 2′-OMe substitution. In contrast, the switch from 2′- to 3′-endo sugar pucker in DNA-based ligands means that such substitutions can be tolerated at positions where ribose conformation is not important or where it can be compensated for by other changes in the molecule. Empirically, at many positions such substitutions perturb the DNA-based SOMAmer structure to reduce the binding affinity to IL-6.

Another substitution that is resistant to endonuclease attack is a C3-spacer. Although this 3-carbon methylene linker preserves the inter-nucleotide spacing, it lacks a sugar and base and is therefore a more drastic change at any single position of the SOMAmer than a 2′-OMe substitution. The C3-spacer unlocks the conformational restriction imposed by the cyclic deoxyribose thus providing a large degree of additional conformational flexibility through fully rotatable bonds in the inter-nucleotide linkage. We also surveyed each dA, dC, and dG position of SL1023 with a C3-spacer substitution and identified a region spanning positions C18–G21 that tolerated this substitution. This region is likely to be spatially distinct from the core-binding elements and not in direct contact with IL-6.

SOMAmers are selected from libraries containing a single dU modification. These modifications are essential components of the intermolecular interaction with the target protein, and although they are acceptable solutions, they may not be ideally complementary to the interaction surface. Additional or fewer linker carbons or planar hydrophobic rings might allow one or more benzyl or naphthyl adducts to adopt a more favorable orientation resulting in improved affinity (32). With this goal in mind, we surveyed each dU position of SL1023 with alternative modifications or no modification (dT), but we observed only modest affinity improvements at only a few positions. However, when we tested combinations, we found the Pe-dU and Nap-dU substitutions at positions 9 and 12, paired with the six 2′-OMe additions in SL1025, resulted in improved inhibition activity and serum stability *in vitro*. One serendipitous outcome of this approach was an increase in percent inhibition achieved with the substitution of Nap-dU at position 12 of SL1025. It is possible that the additional ring of the naphthyl group (compared with the benzyl group) helps to form a more extensive contact surface with IL-6 thereby further stabilizing the complex.

Optimization of Nap-dU SOMAmer SL1030 was performed with a similar strategy. Combinations of C3-spacer substitutions resulted in SL1032, with three C3-spacers in the truncated 30-mer providing a 30-fold improvement in inhibition activity. Perhaps removal of these nucleotides relaxed a structural constraint or facilitated a new interaction with IL-6. Combinations of the three C3-spacer substitutions in SL1032 with 2′-OMe substitutions at tolerated positions have not been extensively evaluated but may offer additional nuclease protection.

The optimization process led to improvements in the binding affinities of SL1025 and SL1032 for human IL-6. SPR analysis revealed that the high affinities can be attributed, at least in part, to exceptionally slow complex dissociation rates, an intrinsic property of SOMAmers. SL1025 exhibited simple monophasic kinetics with a dissociation half-life of 6.9 h, whereas SL1032 showed biphasic kinetics with a fast-dissociating species (5-min half-life) and a slow-dissociating species (28-h half-life). This biphasic behavior may be due to the existence of two kinetically trapped conformations of SL1032, one with tight IL-6 binding and one with weaker binding.

To study ortholog specificity of the lead SOMAmers, the binding properties of SL1025 and SL1032 to rat, mouse, and monkey IL-6 were profiled. Human and monkey IL-6 have about 97% sequence identity as compared with only 40% with rat and mouse IL-6 (Fig. 6*C*). Neither SOMAmer bound rodent IL-6, which was predicted based on the low amino acid sequence conservation. The binding properties of SL1025 and SL1032 were quite different with regard to monkey IL-6 protein. The binding affinity of SL1025 for monkey IL-6 was 10-fold lower than for the human ortholog, while SL1032 showed no binding to monkey IL-6 protein. Because the two SOMAmers compete with each other for IL-6 binding (data not shown) and block IL-6 binding to IL-6Rα (Fig. 9), they likely bind IL-6 protein at overlapping but nonidentical sites and make contact with different residues. There are six amino acid differences between human and monkey IL-6 (P3L, R16S, Q28H, K46R, E81D, and D140E), and one or more of these may be involved in the interaction of SL1032 with IL-6 (Fig. 6*C*). Differences in glycosylation may also contribute to the reduction in affinity for monkey IL-6, as the monkey IL-6 used in these experiments was glycosylated, while the human IL-6 was not. This effect was observed in inhibition studies where the IC_50_ of SL1025 was higher for glycosylated human IL-6 than its nonglycosylated form, and even higher for glycosylated monkey IL-6 (Fig. 6*B*). Although inhibiting either IL-6Rα or gp130 binding will interfere with the IL-6-signaling pathway, as we show in the accompanying paper (50), blocking both receptor-binding sites may lead to more effective inhibition of IL-6 signaling *in vivo*.

Unmodified DNA and RNA aptamers are rapidly degraded by nucleases present in blood and tissues, resulting in half-lives as short as 2 min (42). RNA aptamers for clinical use are heavily modified at the 2′-position of ribose with fluorine (2′-F), methoxy (2′-OMe), or amine (2′-NH_2_) groups or in the phosphodiester backbone with phosphorothioate groups to achieve suitable *in vivo* stability (28). To determine the effect of the 5-dU modifications and 2′-OMe and C3-spacer substitutions on metabolic stability, we tested the SOMAmers in an *in vitro* serum stability assay. This assay served as a surrogate for an *in vivo* pharmacokinetic evaluation to assess the sensitivity of SOMAmers to endonucleases present in plasma in the absence of other clearance mechanisms. As expected, unmodified DNA control sequences SL1023dT and SL1031dT were rapidly degraded. The benzyl and naphthyl modifications present in SL1023 and SL1031 increased half-life by about 10-fold, indicating that these adducts interfere with substrate recognition by serum nucleases and alone offer a degree of resistance not present in unmodified aptamers. It is also possible that SL1023dT and SL1031dT adopt alternative conformations, exposing endonuclease sites not present on SL1023 and SL1031. Another substantial increase in stability was achieved by the addition of 2′-OMe and C3-spacer modifications in SL1025 and SL1032, indicating our optimization efforts were successful.

In addition to its role in inflammatory disease, IL-6 signaling is also a key component of tumor cell proliferation (43). IL-6 receptor is overexpressed in some cancers, including brain, prostate, and kidney, and elevated IL-6 ligand and receptor expression are associated with poor patient survival. Inhibition of IL-6 signaling may suppress growth, survival, and/or metastatic potential of tumor cells. Inhibition of tumor cell proliferation by SL1026 and SL1033 was demonstrated in U266B1 myeloma, HepG2 hepatoma, and U87MG glioma cells, and the potency of SOMAmer inhibition of IL-6 was equal to or greater than tocilizumab inhibition of IL-6Rα in all cases (Fig. 8).

The role of constitutive activation of the IL-6-signaling pathway is well established in inflammation, and an increase in IL-6 and soluble IL-6Rα in the synovial fluid of joints in rheumatoid arthritis patients was shown to correlate with disease progression (44). The humanized anti-IL-6 receptor antibody tocilizumab has afforded some benefit to patients with rheumatoid arthritis, Castleman disease, and juvenile idiopathic arthritis (45). However, side effects of both classical and trans-signaling inhibition of IL-6Rα by tocilizumab have been reported, including increased cholesterol and triglyceride levels accompanied by weight gain (45–47). Also, an increase in IL-6 protein is seen in patients treated with tocilizumab (46). Direct inhibition of IL-6 may offer some advantages over inhibition of IL-6Rα because of the known role of soluble IL-6Rα in facilitating clearance of IL-6 from the circulation (46). Inhibition of IL-6 with the anti-IL-6 antibody clazakizumab was recently shown to be effective at controlling the symptoms of rheumatoid arthritis in a phase IIb clinical study (48). Clazakizumab was also shown to be more potent than tocilizumab at blocking IL-6 induced cell functions *in vitro* (49).

The IL-6 SOMAmers described in this report have properties well suited for this therapeutic challenge, including high affinity, slow complex dissociation, endonuclease resistance, and potent inhibition of IL-6 signaling. Although these SOMAmers antagonize IL-6 activity *in vitro*, an *in vivo* evaluation in an inflammation model is required to understand the true therapeutic potential of this new class of IL-6 inhibitors.

## Footnotes

2 The abbreviations used are:

SELEX: systematic evolution of ligands by exponential enrichment
Bn-dU: 5-(*N*-benzylcarboxamide)-2′-deoxyuridine
Nap-dU: 5-[*N*-(1-naphthylmethyl)carboxamide]-2′-deoxyuridine
Pe-dU: 5-[*N*-(phenyl-2-ethyl)carboxamide]-2′-deoxyuridine
Pp-dU: 5-[*N*-(phenyl-3-propyl)carboxamide]-2′-deoxyuridine
2′-OMe: 2′-methoxy
SPR: surface plasmon resonance.
